# 

**Published:** 1895-01-12

**Authors:** 


					tke hospital.
/o
i
o PRICE TWOPENCE.
No. 433, Vol. XVII-Jan. 12th, 1895.
^B^gistered at the General Post Office as a Newspaper.
Jan. 12, 1895.
WITH EXTRA NURSING SUPPLEMENT.
Per Annum, 10s, 6d., post free.
Monthly Farts, 6d.; post firee, 8d.
Ditto per Annum, 8s 6d. post free.
lAsootr Western Infirmary
No. 433. Vol. XYII.
HDSP/7
WITH EXTRA NURSING SUPPLEMENT.
'RWICH HOSP l.TAJ.
Saturday, Jan. 12th, 1895.

				

